# Microcalorimetric study of the growth of *Streptococcus thermophilus* in renneted milk

**DOI:** 10.3389/fmicb.2015.00079

**Published:** 2015-02-10

**Authors:** Irina Stulova, Natalja Kabanova, Tiina Kriščiunaite, Kaarel Adamberg, Tiiu-Maie Laht, Raivo Vilu

**Affiliations:** ^1^Department of Chemistry, Tallinn University of TechnologyTallinn, Estonia; ^2^Competence Centre of Food and Fermentation TechnologiesTallinn, Estonia

**Keywords:** *Streptococcus thermophilus*, growth and metabolism of the bacteria, milk, renneted milk, microcalorimetry

## Abstract

The growth of *Streptococcus thermophilus* ST12 (ST12) in liquid milk, reconstituted from low-heat skim milk powder reconstituted skim milk (RSM) and in RSM with rennet addition (r-RSM) at 40°C was monitored by microcalorimetry. It was shown that the growth rate of bacteria decreased in renneted samples in comparison with liquid RSM starting from certain sizes of the colonies (“deviation moments”), which depended on the inoculation rates. The hydrolysis of lactose was delayed for about 1 h in the r-RSM in comparison with RSM but otherwise the metabolism of carbohydrates in the renneted and non-renneted milks was similar. The total free amino acids (TFAA) content by the end of fermentations was higher in r-RSM than in RSM presumably due to the enzymatic hydrolytic activity of rennet. The quantitatively dominating amino acids were remarkably different in the r-RSM and RSM indicating that the hydrolysis cascade of caseins and/or metabolism of amino acids by the bacteria functioned differently in the two cases. The data obtained showed potential of microcalorimetry to characterize quantitative differences of growth and metabolism of the bacteria in renneted and liquid samples of milk.

## Introduction

Studies of growth of LAB in the liquid milk (Favrot and Maubois, 1994, 1996; Letort et al., 2002) and solid cheese are complicated because traditional microbiological methods are not well suited for the enumeration of bacteria in opaque and especially in solid media. Peculiarities of colonial growth of bacteria in solid transparent matrices (agar, gelatine) have been described, however, in several papers (Brocklehurst et al., 1995; McKay et al., 1997; Malakar et al., 2002; Wilson et al., 2002; Kabanova et al., 2009, 2012; Velliou et al., 2013). Studies of growth of LAB in solid coagulated milk samples are clearly less numerous (Favrot and Maubois, 1996; Floury et al., 2010, 2013; Jeanson et al., 2011). One of the reasons of the noted scarcity is the lack of suitable methods for the studies.

The most noticeable advantage of the calorimetric method in comparison with the other mostly invasive techniques is the possibility to follow the bacterial growth in opaque and/or solid environments like various food matrices monitoring the heat evolution without destroying or perturbing samples studied (Lobete et al., in press). The calorimetric technique was first used in dairy research in seventies for milk grading (Berridge et al., 1974) and growth studies of pure LAB cultures under limiting substrate conditions (Fujita et al., 1978). The interactions of *Streptococcus thermophilus* and *Lactobacillus bulgaricus* were studied in milk using calorimetry in 1979 (Monk, 1979). Riva et al. (1997) investigated shelf life of fresh milk using isothermal calorimetry for continuous monitoring of microbial growth. Gardea et al. (2002) compared heat evolution by bacteria with the results of traditional plate counts assessing microbiological quality of milk undergone different treatments. Wadsö and Galindo (2009) compared the thermal power-time profiles of the fermentation of milk at two incubation temperatures using two different buttermilk cultures. Kriščiunaite et al. (2011) applied microcalorimetry to study the influence of H_2_O_2_ on the growth of thermophilic starter bacteria in UHT milk.

A considerable methodical improvement in use of microcalorimetry was introduced in Kabanova et al. (2009) and Kabanova et al. (2012). Two major advancements introduced were as follows: (a) a method of serial dilution was developed, which means that inoculation rates of the bacteria in the samples were changed from 10^0^ to 10^6^ CFU mL^−1^ with the precisely ten-fold increment increment. This allows to study the growth of the populations and colonies of different numbers of cells, and (b) a number of additional samples prepared in parallel with the microcalorimetric samples are incubated at the same temperature, and they are used for the measurements of sugars, organic and amino acids etc. The data obtained from the study of these parallel samples is used together with the microcalorimetric data. The approach was applied for the characterization of peculiarities of the growth of *Streptococcus thermophilus* ST12 in differently pretreated milk samples (Stulova et al., 2011), and in milk reconstituted from non-irradiated and irradiated at 10 kGy milk powder (Stulova et al., 2013).

The approach developed was used also in this paper. The specific aim of the present study was to investigate the growth of *Streptococcus thermophilus* ST12 in renneted reconstituted skim milk (RSM) (in milk gel) and compare the peculiarities of the growth with those in liquid milk samples.

## Materials and methods

### Preparation of reconstituted skim milk (RSM)

Low heat skim milk powder (LHSMP) was obtained from Valio Ltd. (Helsinki, Finland). LHSMP was suspended in distilled water to yield a final concentration of 10% (w v^−1^) milk solids, with addition of CaCl_2_ in final concentration of 10 mM. The samples with LHSMP were mixed thoroughly for 1 h at room temperature, heated at 90°C for 30 min and cooled to 30°C immediately before the experiments and used as liquid RSM samples.

### Bacterial cultures and preparation of inoculum

The strain of *Streptococcus thermophilus* ST12 (further *St. thermophilus* ST12) was kindly provided by Chr. Hansen (Hørsholm, Denmark). Deep-frozen cultures of *St. thermophilus* ST12 were thawed and pre-grown on M17 agar (LAB M, Lancashire, UK) for 24 h at 40°C. One colony from a M17 agar plate was inoculated into 10 mL of RSM and left at 40°C until coagulation (16 h). One per cent of the culture was used for inoculation of the 10 mL of RSM samples, left until coagulation (12 h) and further used for inoculation of the microcalorimetry samples. The number of viable cells in the inocula was determined by plating on M17 agar followed by incubation for 72 h at 40°C to be (1.15 ± 0.19) × 10^9^ CFU mL^−1^.

### Preparation of samples for growth experiments

RSM as growth media were prepared in 50 mL Erlenmeyer flasks. Milk samples were inoculated with 1% (v v^−1^) of inoculum and stirred thoroughly. The concentrations of inocula in milk samples for microcalorimetry were varied from 10^1^ to 10^6^ CFU mL^−1^ with the 10-fold increment. Milk gels were prepared by addition of 100 μl of a 10 g L^−1^ chymosin (CHY-MAX Powder Extra, Hørsholm, Denmark) aqueous solution to 10 mL of RSM. The enzyme (chymosin) was added immediately after the inoculation. Milk samples for the microcalorimetric experiments, 2 mL samples of RSM and r-RSM were transferred into the autoclaved microcalorimetric ampoules, inserted into microcalorimeter and maintained at 40°C. Monitoring of the growth of microorganisms was started in microcalorimeter about 1 h after the inoculation, after the temperature of the samples was in perfect equilibrium with the set temperature of the thermostat (40°C). Taking into account that the coagulation of the liquid milk samples was over in 40 min after the inoculation, the growth of the bacteria in case of the r-RSM samples was studied in practice in solid gel matrices from the beginning of the microcalorimetric monitoring.

The remaining after the preparing of the microcalorimetric samples inoculated and renneted milks were divided into 1 mL aliquots and incubated at 40°C in parallel with the samples in microcalorimeter and used for the determination of pH and the concentrations of carbohydrates, lactic acid and amino acids at appropriate time points, determined based on the peculiarities of power-time curves.

### Microcalorimetry

A 24 channel isothermal batch microcalorimeter TAM III Thermal Activity Monitor (TA Instruments, New Castle, DE, USA) was used for the monitoring of the growth of thermophilic starter *St. thermophilus* ST12. Data acquisition and analysis was carried out using TAM Assistant Program (v 0.9.1012.40, SciTech Software AB, Thermometric AB).

#### Analysis of calorimetric power-time curves and calculation of growth characteristics

**Figure 1:**
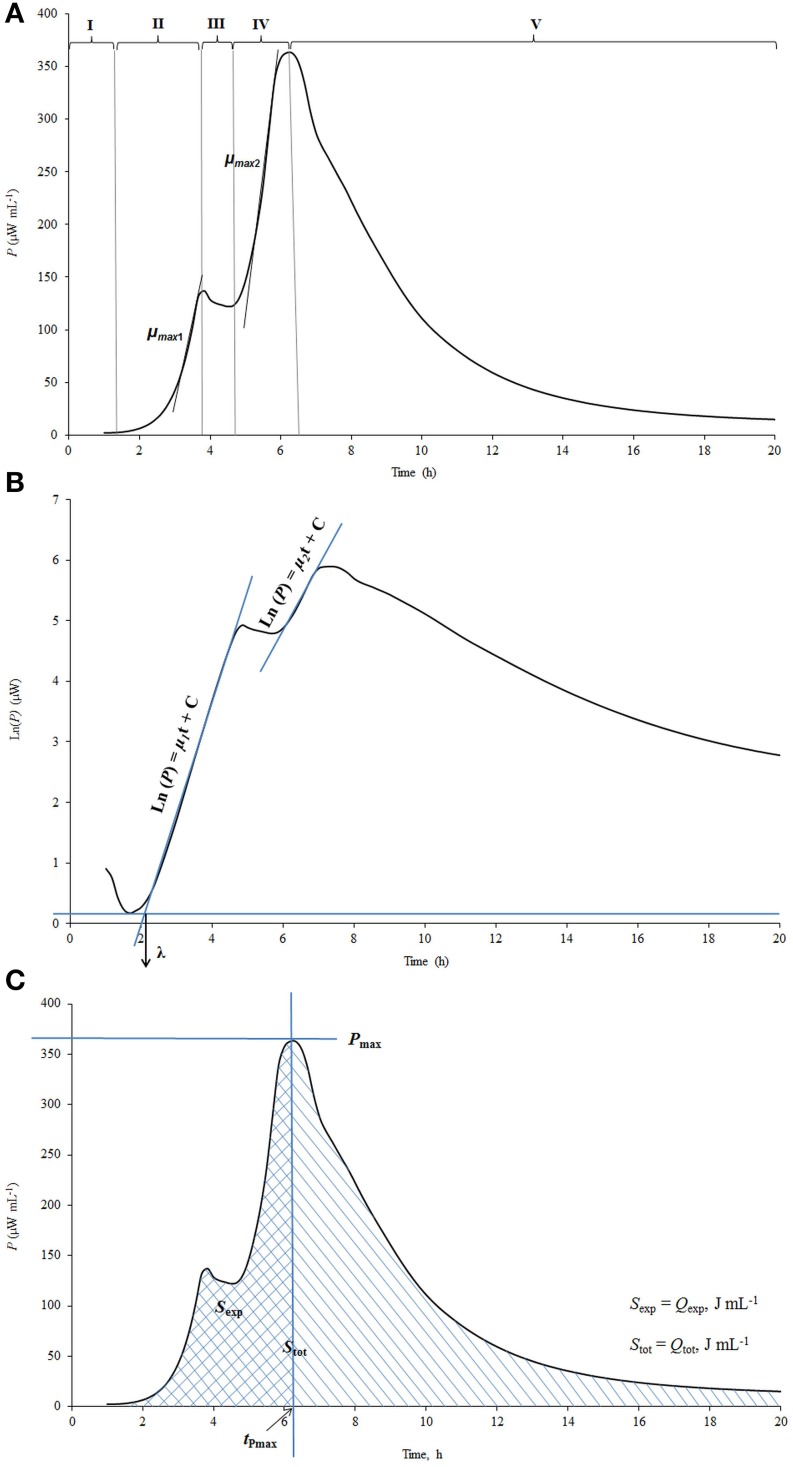
**The explanation of the processing of calorimetric power-time curves. (A)** The division of calorimetric power-time curves into five phases; **(B)** Determination of the maximal calorimetric growth rates during the first (μ_max1_, Wh^−1^) and the second exponential phase (μ_max2_, Wh^−1^), and the duration of the lag-phase (λ, h); **(C)** Determination of the heat evolution during combined exponential phases (*Q_exp_*), and during the growth of the cultures (*Q_tot_*).

The power-time curves, the output of microcalorimeter describe the heat evolution during the processes studied. A calorimetric power-time curve of the growth of *St. thermophilus* ST12 in milk measured by us (Stulova et al., 2013) was divided into five different growth phases as shown in Figure 1A.

The pregrowth phase after the inoculation in the beginning of the graph is characterized as the lag-phase (I) during which bacterial cells adapt to the new environment. The length of the lag-phase was determined as shown on Figure 1B, where the power-time curve is presented in semilog scale. It is important to note that in measuring of the length of the lag-phase on the basis of calorimetric curves the sensitivity of the instrument TAM III (7 × 10^−4^ J/0.5 μW) as well as the time during which heat produced by the cells exceeds the level of sensitivity of the instrument should be taken into account (see Kabanova et al., 2009, 2012). The second phase of the power-time curves following the lag-phase corresponds to the first exponential growth phase (II) during which the bacteria grow at the maximal calorimetric growth rate μ_max_ possible in the experimental conditions studied. During the intermediate non-exponential growth phase (III) lactic acid bacteria presumably synthesize proteinases and metabolism of this bacteria switch from growth on free amino acids (FAA) present in milk to growth on amino acids and peptides released by the enzymes synthesized (Letort et al., 2002). The ends of the first as well as the second exponential phases (IV) of bacterial growth were defined by the corresponding peaks of the power-time curves. In case of analysis of certain metabolic characteristics of the bacteria during the growth (consumption patterns of amino acids etc.) the two exponential phases were considered as one—see below. After the second exponential growth phase the deceleration phase (V) which combines the stationary phase of the growth together with the deceleration of the metabolism of lactic acid bacteria growing in milk was taking place.

Maximum specific growth rate (μ_max_), which could be considered also as maximal calorimetric growth rate during the first and second exponential growth phases was measured as shown in Figure 1B. Taking into account that during the exponential growth phase the relationship between biomass concentration (*X*) and specific growth rate (μ) may be described by the first order kinetics:

(1) $$\frac{dX}{dt}=\mu X$$

and assuming that the rate of biomass formation (d*X*/d*t*) was proportional to the rate of heat production (d*Q*/d*t*), the maximum specific growth rate (μ_max_) was calculated from the power-time curves as a slope of ln d*Q*/d*t* over time (*t*), as shown in Maskow and Babel (2003):

(2) $$\ln\frac{dQ}{dt_{t}}=\frac{dQ}{dt_{0}}+\mu_{\max}t$$

The heat produced during the exponential growth phase *Q*_exp_ (area between the power-time curve and the vertical line that goes through the major peak of the power-time curve (*P*_max_ value) and the baseline—see Figure 1C and the total heat produced during the whole process of growth *Q*_tot_(area between the power-time curve and baseline) were determined using the TAM Assistant program (v 0.9.1012.40, SciTech Software AB, Thermometric AB, Järfälla, Sweden) and Microsoft Excel. The recording of the heat evolution was taking place with the frequency 1 s^−1^. The time-points were resampled at equidistant intervals of 15 min.

The value *P*_max_ is characterizing the maximum aggregate rate of heat production (see Figure 1C). After reaching the maximum, the decrease of heat production rate starts. The time point where *P*_max_ is measured is a convenient time for the end of the exponential growth phase(s).

The change of the number of viable cells over time was calculated using the equation (Stulova et al., 2013):

(3) $$N=\frac{\Delta Q}{YQ}$$

where *N* (CFU mL^−1^ h^−1^) is the number of bacteria grown during the selected time interval, *ΔQ* (J mL^−1^ h^−1^) is the heat produced during the same time interval, and *Y_Q_*(J CFU^−1^) is the experimentally determined heat yield coefficient (*Y_Q_*, J CFU^−1^). Heat yield coefficient (*Y_Q_*, J CFU^−1^) was calculated on the basis of the results obtained from the experiments of ST12 growth in liquid RSM. The plate count was carried out at the end of exponential growth phase (6.31^*^10^8^ CFU mL^−1^) and heat amount produced was calculated from the power-time curves (2.81 J). The value of *Y_Q_* (J CFU^−1^) was determined to be *Y_Q_* = 2.81 J/6.31^*^10^8^ CFU mL^−1^ = 4.45^*^10^−9^ J CFU^−1^. Our earler experiments (unpublished data) showed that the *Y_Q_* value depended remarkably on the temperature of the growth but not on the media composition or inoculation rate. The *Y_Q_* value indicated was used for the description of growth in the liquid cultures (RSM) as well as in the case of solid-state growth (in r-RSM).

The numbers of bacteria at the end of exponential growth phase (*N*_exp_, CFU mL^−1^) and the total numbers grown during the whole process (*N*_tot_, CFU mL^−1^), and also at “deviation moments” (N^*^, CFU mL^−1^)—see below, were calculated from heat evolution data *Q*_exp_(J mL^−1^) and *Q*_tot_ (J mL^−1^), and Q^*^ (J mL^−1^) using the indicated above *Y_Q_* value (Kabanova et al., 2012).

The radii of average spherical model colonies (*R*_col_, μm) were calculated from average volumes of colonies using Kepler's conjecture of packing of determined by heat measurements numbers of bacteria in colonies (Hsiang, 1993; Hales, 1994)—see a detailed discussion of the use of the conjecture in Kabanova et al. (2012).

### Analytical methods

Incubated in parallel to the microcalorimetric samples milk samples were mixed at chosen times 1:1 with isopropanol for sedimentation of proteins. The precipitate was removed by centrifugation at 14,000 × g for 10 min. The supernatant was filtered through a 13 mm diameter and 0.2-μm pore-size regenerated cellulose (RC) membrane filter (Whatman, Maidstone, UK) and diluted with water before analysis. High-performance liquid chromatography (HPLC) system (Alliance 2695 system, Waters Corp., Milford, MA) with a Refractive Index Detector 2414 and column BioRad HPX-87H 300 × 7.8 mm (Hercules, CA) was used for measuring lactose, glucose, galactose and lactate concentrations.

Analysis of FAA was performed on an ultra-performance liquid chromatography (UPLC) system (Acquity UPLC; Waters Corp.) including a binary solvent manager, a sample manager and photodiode array (PDA) detector, connected to Waters Empower™ 2.0 software. Separations were performed on a 2.1 × 100 mm Waters Acquity UPLC AccQ•Tag Ultra Column operated at 55°C, the running time being 12 min. Sample derivatization procedure was as follows: 20 μl AccQ•Fluor reagent was added to the mixed solution of 70 μl AccQ•Fluor borate buffer and 10 μl sample or standard. The mixed solution was vortexed immediately for 10 s, transferred to an autosampler vial and allowed to stand at room temperature for 1 min. Then the vials were placed in a heating block at 55°C for 10 min, after which they were analyzed by the UPLC system. Empower software (Waters Corp.) was used for the data processing.

The pH of milk samples was measured with pH meter S20 Seven Easy equipped with InLab 413 electrode (Mettler-Toledo GmbH, Greifensee, Switzerland).

### Calculation of growth parameters characterizing metabolism of the growing cells

The growth characteristics of the bacteria (yield coefficient values, growth rates etc.) during the growth in milk were calculated on the basis of the heat evolution converted to biomass concentrations or cell numbers and concentrations of organic acids etc. in the culture media as follows:

(4) $$\mu =\frac{\ln\;(N_{2}/N_{1})}{t_{2}-t_{1}}$$

(5) $$I_{\text{Lactose}}=\frac{d(C_{\text{Lactose}})}{d(X)}$$

(6) $$O_{\text{Lactose}}=\frac{d(C_{\text{Lactose}})}{d(X)}$$

(7) $$Y_{\text{LactateLactose}}=\frac{d(C_{\text{Lactate}})}{d(C_{\text{Lactose}})}$$

(8) $$Y_{XS}=\frac{d(X)}{(d(2\cdot C_{\text{Lactose}})-d(C_{Gal})-d(Glc))\times 180/1000}\;$$

(9) $$I_{AA}=\frac{d(C_{AA})}{d(X)}$$

where μ is the specific growth rate (h^−1^), t is time (h), *N* is the number of cells calculated assuming that heat produced per cell formation is constant (*Y_Q_* = 4.45 × 10^−9^ J per cell), *X* is the dry biomass (gdw L^−1^), assuming that the mass of one cell is 0.2 × 10^−12^ g (unpublished data), I_Lactose_ is the lactose consumption per biomass produced (mmol gdw^−1^), *O*_Lactate_ is the lactate production per biomass produced (mmol gdw^−1^); C designates the concentration of corresponding compound, for example *C*_Lactate_ (mM), *Y*_Lactate/Lactose_ shows the lactate yield per lactose consumed, *Y*_XS_ is the biomass yield per hexose consumed and *I*_AA_ is the consumption of corresponding amino acid per biomass produced (mmol gdw^−1^).

### Statistical analysis of the data

All the microcalorimetric experiments were repeated twice and measurements were carried out with two or three parallel samples. The power-time curves presented on the Figures and used in the calculations are combined curves of two parallel experiments.

The other analyses (HPLC, UPLC, pH) were carried out in triplicate. All values of the parallel experimental points were averaged and reported along with the value of standard deviation (SD). The experimental data were submitted to single-factor analysis of variance (ANOVA), and the differences of the means were evaluated by Fisher's least significant difference (LSD) test. The difference of the mean values was accepted at the significance level *p < 0.05*. The Student's *t*-test was performed to evaluate statistically significant differences between the mean calorimetric values obtained in RSM and r-RSM.

## Results

### Peculiarities of the growth of *St. thermophilus* ST12 in r-RSM

**Figure 2:**
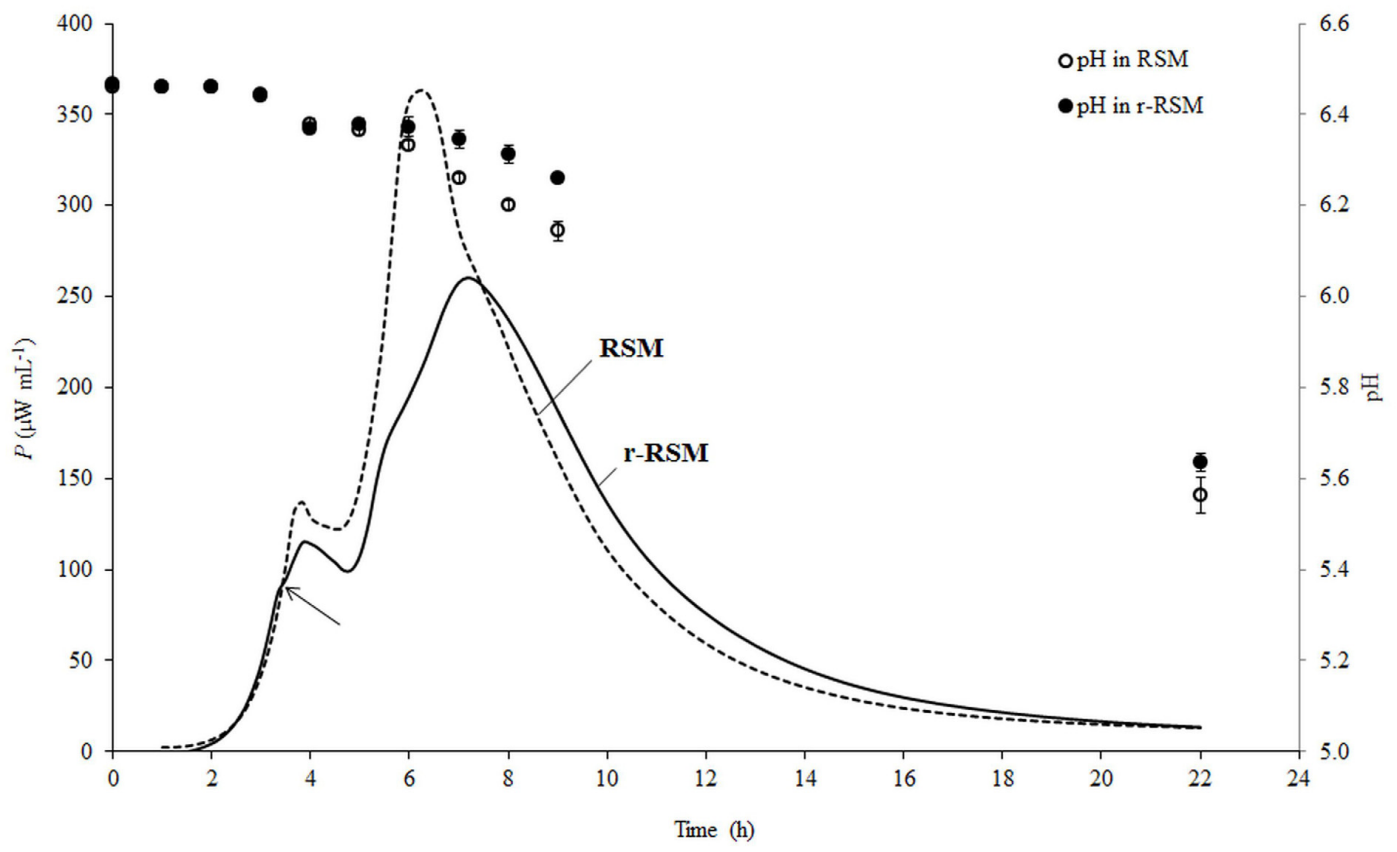
**Calorimetric power-time curves of the growth of *Streptococcus thermophilus* ST12 and pH changes (circles) in liquid (dashed) and renneted (black) reconstituted skim milk at inoculation rate 10^5^ CFU mL^−1^**. Arrow is indicating the deviation moment where the growth rate in r-RSM is decreasing in comparison with the RSM.

Representative calorimetric power-time curves of the growth of ST 12 in RSM and r-RSM, at inoculation rate 10^5^ CFU mL^−1^, are presented in Figure 2.

As was previously described (Stulova et al., 2013), a typical microcalorimetric power-time curve of inoculated RSM contained two peaks corresponding to two distinctively separated growth phases (first and second exponential growth phases). As seen in Figure 2, the general pattern with two major exponential growth phases was observed also in r-RSM. The growth curves of *St. thermophilus* ST12 in r-RSM and RSM were practically coinciding in the beginning, but starting from a certain number of the bacteria in the sample (N^*^ = 0.63 × 10^7^ CFU mL^−1^ at *N*_0_ = 10^5^ CFU mL^−1^), at the “deviation moment” marked by the arrow in Figure 2 the maximal calorimetric growth rate (μ_max1_) in r-RSM was decreased, branching from the curve of RSM, and the further growth in the coagulated matrix was clearly different from that in RSM. This led to the appearance of the second slower phase in the first exponential phase in comparison with the growth curve in milk (RSM). The splitting of the second exponential growth phase also into two was observed as well—see Figure 2.

**Figure 3:**
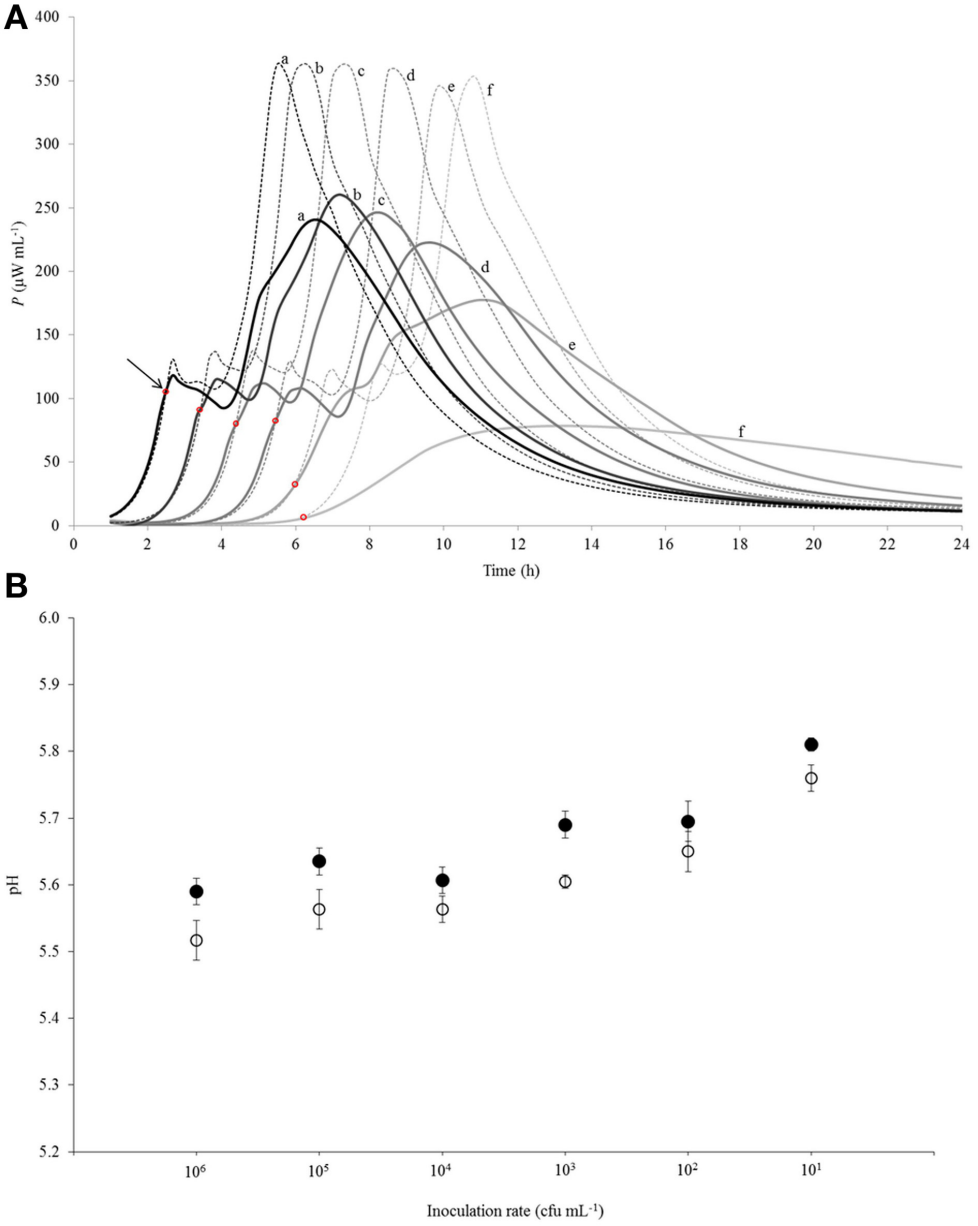
**(A)** Calorimetric power-time curves describing growth of *Streptococcus thermophilus* ST12 in liquid (dashed line) and renneted (bold line) reconstituted skim milk at different inoculation rates (CFU mL^−1^): (a) 10^6^; (b) 10^5^; (c) 10^4^; (d) 10^3^; (e) 10^2^; (f) 10^1^. The arrow and the red circles mark the “deviation moments.” **(B)** pH at the end of fermentation (at 22 h) at all inoculation rates (10^6^–10^1^ CFU mL^−1^) in RSM (white circles) and in r-RSM (black circles). Initial pH = 6.47 was the same in the samples.

As seen in Figure 2, acidification developed more slowly after 3.5 h of incubation in renneted milk compared to the liquid milk samples which was in a good correlation with the slower growth rate of the bacteria in r-RSM. Higher pH values in r-RSM samples accompanying the main part of the growth of the bacteria were observed in case of all inoculation rates studied (Figure 3B), in difference from a previous report (Favrot and Maubois, 1996) showing slower acidification in rennet curd only at lower level of inoculation (10^2^ CFU mL^−1^).

#### Effect of inoculation rate on power-time curves of *St. thermophilus* ST12 in r-RSM

Calorimetric power-time curves of the growth of ST 12 in RSM and r-RSM from 10^6^ to 10^1^ CFU mL^−1^ with increments of 10-fold, are presented in Figure 3 and processed results were summarized in Table 1. The *t*-test results denoting the differences between liquid and renneted RSM are presented in Table 2.

**Table 1:** **Growth characteristics of *Streptococcus thermophilus* ST12 in RSM and r-RSM at 40°C^a^**.

**Milk**	**Inoculation rate (cfu mL^−1^)**	**μ_max_(Wh^−1^)**		**Q_1−exp.ph_ (J mL^−1^)**	**Q_exp.ph_ (J mL^−1^)**	**Q_tot_ (J mL^−1^)**	**P_max_ (μW)**	**t_Pmax_ (h)**	**λ (h)**
		**μ_max1_**	**μ_max2_**						
RSM	10^6^	1.88, 0.03^aA^	1.05, 0.01^aA^	0.28, 0.00^aA^	2.00, 0.05^aA^	7.12, 0.01^aAB^	362.95, 3.80^aA^	5.50, 0.00^aA^	0.02, 0.00^aA^
	10^5^	1.85, 0.05^aA^	1.18, 0.00^bA^	0.32, 0.00^bA^	2.10, 0.00^bA^	7.21, 0.03^aA^	362.77, 4.03^aA^	6.17, 0.00^bA^	0.98, 0.05^bA^
	10^4^	1.91, 0.03^aA^	1.17, 0.02^bA^	0.34, 0.00^bA^	2.11, 0.01^bA^	7.12, 0.01^aA^	363.10, 0.47^aA^	7.33, 0.00^cA^	2.08, 0.02^cA^
	10^3^	1.85, 0.04^aA^	1.14, 0.01^bA^	0.32, 0.01^bA^	1.97, 0.02^aA^	7.24, 0.02^aA^	359.57, 2.30^aA^	8.67, 0.00^dA^	3.05, 0.05^dA^
	10^2^	1.83, 0.10^aA^	1.13, 0.02^bA^	0.35, 0.04^bA^	2.00, 0.07^aA^	7.23, 0.07^aA^	345.52, 8.05^aA^	9.92, 0.12^eA^	4.07, 0.19^eA^
	10^1^	1.61, 0.04^bA^	1.01, 0.03^a^	0.41, 0.02^c^	2.26, 0.02^cA^	7.36, 0.13^aA^	353.31, 12.09^aA^	10.83, 0.00^fA^	4.98, 0.01^fA^
r-RSM	10^6^	1.89, 0.01^abA^	1.00, 0.01^aA^	0.28, 0.00^aA^	2.41, 0.04^aB^	7.02, 0.02^aB^	240.67, 4.78^abB^	6.50, 0.00^aB^	0.04, 0.00^aA^
	10^5^	2.01, 0.15^aA^	1.06, 0.01^aB^	0.33, 0.04^bA^	2.36, 0.07^aB^	6.92, 0.40^aA^	260.32, 0.37^aB^	7.17, 0.00^aB^	1.15, 0.27^bA^
	10^4^	1.89, 0.02^abA^	0.94, 0.10^aB^	0.45, 0.00^bB^	2.26, 0.20^aA^	6.89, 0.31^aA^	247.32, 19.66^aB^	8.17, 0.23^bB^	1.96, 0.02^cB^
	10^3^	1.88, 0.04^abA^	0.93, 0.00^aB^	0.44, 0.01^bB^	2.28, 0.00^aB^	7.12, 0.07^aAB^	222.73, 2.02^bB^	9.59, 0.12^cB^	2.97, 0.04^dA^
	10^2^	1.67, 0.09^bA^	0.49, 0.03^bB^	0.55, 0.01^cB^	2.45, 0.11^aB^	7.35, 0.56^aA^	177.90, 1.63^cB^	11.00, 0.24^dB^	3.87, 0.07^eA^
	10^1^	1.12, 0.01^cB^	–	–	1.37, 0.32^bB^	6.47, 0.06^aB^	78.75, 6.77^dB^	13.25, 0.59^eB^	4.64, 0.15^fB^

a *Data are means ± SD of maximal calorimetric growth rate (μ_max_) in the first and second exponential growth phase, the heat evolved during the first and second exponential phase (Q*_1_*_stexp.ph_ and Q_exp.ph_), the total heat produced during the whole fermentation (Q_tot_), maximum heat flow (P_max_), the time of the maximum heat production rate (t_Pmax_) and lag phase duration (λ) obtained from microcalorimetric power-time curves*.

**Table 2:** **The *t*-test analysis denoting the differences between liquid and renneted RSM samples**.

**Milk**	**Inoculation rate (cfu mL^−1^)**	**μ_max_ (Wh^−1^)**		**Q_1−exp.ph_ (J mL^−1^)**	**Q_exp.ph_ (J mL^−1^)**	**Q_tot_ (J mL^−1^)**	**N_exp (cfu mL^−1^)_**	**P_max_(μW)**	**t_Pmax (h)_**	**λ (h)**
		**μ_max1_**	**μ_max2_**							
RSM vs. r-RSM	10^6^	NS	^*^	NS	^*^	^*^	^*^	^**^	^***^	NS
	10^5^	NS	^**^	NS	^*^	NS	^*^	^***^	^***^	NS
	10^4^	NS	^**^	^**^	NS	NS	^*^	^*^	^*^	^*^
	10^3^	NS	^**^	^**^	^**^	NS	^**^	^***^	^**^	NS
	10^2^	NS	^**^	^**^	^*^	NS	^*^	^**^	^*^	NS
	10^1^	^**^	–	–	^*^	^*^	^*^	^**^	^*^	NS

*NS—not significant*,

* *P < 0.05*,

** *P < 0.01*,

*** *P < 0.001*.

According to Table 2, the first exponential growth phases of *St. thermophilus* ST12 in r-RSM were shorter and the calculated μ_max_ values were higher than in the second phase like in the case of RSM, however, the noted differences were much more strongly expressed (see also Stulova et al., 2013). As seen from the data presented in Tables 1, 2 the maximal calorimetric growth rates of the first exponential phase (μ_max1_) were similar (*P* > 0.05) in RSM and r-RSM at different inoculation rates ranging from 10^6^ to 10^2^ CFU mL^−1^, except 10^1^ CFU mL^−1^. The values of maximal calorimetric growth rates of the second exponential phase (μ_max2_) in r-RSM were significantly lower (*P <* 0.01) than in RSM. This is indicating that the growth of the bacteria in bigger colonies in case of r-RSM is most probably inhibited by the accumulating lactate.

The integrating of the power-time curves allows measure the heat produced by the growing bacteria, where *Q*_exp_ indicates the heat produced during the exponential growth phase and *Q*_tot_ shows the total heat produced by the total biomass formed during fermentation. According to Table 1 the amounts of heat produced during the exponential phase (*Q*_exp_, _average_) in r-RSM at inoculation rates ranging 10^6^–10^2^ CFU mL^−1^ were practically the same with average 2.35 J mL^−1^ which corresponds to 5.30 × 10^8^ CFU mL^−1^ (calculated using heat yield coefficient *Y*_Q_). It was about 13% higher than the same value in RSM (2.04 J mL^−1^). This fact showed that during the exponential growth phase more bacteria were produced in r-RSM. Noting that the amount of lactate produced during the exponential growth was practically the same in the samples this fact allows to assume that pH was most probably the factor determining the end of the exponential growth. The amount of heat produced during the total growth (*Q*_tot_) was the same in r-RSM and RSM at inoculation rates ranging 10^5^–10^2^ CFU mL^−1^−−7.14 ± 0.09 J mL^−1^ that corresponds to the number of bacteria 1.62 × 10^9^ CFU mL^−1^. The *Q*_tot_ and *N*_tot_ values were the lowest in r-RSM at inoculation rate 10^1^ CFU mL^−1^−−6.47 ± 0.06 J mL^−1^ and 1.45 × 10^9^ CFU mL^−1^ respectively. This fact showed that growth of the large colonies was clearly inhibited in coagulated milk (but the same amount of bacteria grew without inhibition in RSM).

Clear differences in the *P*_max_ values between different inoculation rates in r-RSM are shown in Figure 3. The *P*_max_ values were clearly different also if r-RSM and RSM values were compared with each other. As shown in Stulova et al. (2013) the values of *P*_max_ were much higher (in average 350 μW mL^−1^) and very similar at different inoculation rates (see Figure 3). The time to reach the maximum heat effect (*t*_Pmax_) increased with decreasing amounts of inocula (Table 1). As seen from Table 2, there were significant differences noted in *t*_Pmax_ between r-RSM and RSM samples. Depending on the inoculation rate, 1–2.5 h more time was required to reach the maximum heat production rate (*t*_Pmax_) in r-RSM compared with RSM (Table 1). These results showed that together with the decrease of the growth rate the length of the exponential phase was increased, which was the expected result.

The most likely explanation for the inhibition of *St. thermophilus* ST12 growth in r-RSM is that lactate accumulating in the colonies during growth in r-RSM was changing the pH locally and this led to the inhibiton of growth. The peculiarities of colonial growth of bacteria will be discussed in more detail below (in section Discussion).

Lengths of the lag-phases (λ) in r-RSM, as in the case of RSM were changing with regular interval from one inoculation rate to another and became longer on decreasing of the initial inoculum. According to the statistical analysis (Table 2), no big differences were observed in lag-phase (λ) durations at the same inoculation rates in both media studied.

#### Characterization of growth of colonies

Numbers of bacteria in average colonies in case of different inoculation rates were calculated using the data of the heat evolved during the different phases of the growth and *Y_Q_* value. The radii of the average colonies were calculated using Kepler's conjecture of bacterial packing in colonies (Hsiang, 1993; Hales, 1994). The numbers of bacteria in average colonies (CFU/col) and mean sizes of the average colonies (*R*_col_, μm) at the “deviation moments,” at the end of exponential growth phase and at the end of fermentation at different inoculation rates (from 10^6^ to 10^1^ CFU mL^−1^) in r-RSM are presented in Tables 3, 4, respectively.

**Table 3:** **The numbers of bacteria in the sample (cfu mL^−1^) and in average colonies (cfu col^−1^) at the “deviation moment,” at the end of exponential growth phase and at the end of cultivation at different inoculation rates from 10^6^ to 10^1^ cfu mL^−1^ in r-RSM**.

**Inoculation rate, cfu mL^−1^**	N^*^ at power-time curves “deviation moment”		**N_exp_ at the end of exponential phase**		**N_tot_ at the end of fermentation (at 22 h)**	
	**cfu mL^−1^, ×10^7^**	**cfu col^−1^**	**cfu mL^−1^, ×10^8^**	**cfu col^−1^**	**cfu mL^−1^, ×10^9^**	**cfu col^−1^**
10^6^	6.23 ± 0.33	(6.23 ± 0.33) × 10^1^	5.42 ± 0.08	(5.42 ± 0.08) × 10^2^	1.58 ± 0.01	(1.58 ± 0.01) × 10^3^
10^5^	4.96 ± 0.24	(4.96 ± 0.24) × 10^2^	5.30 ± 0.16	(5.30 ± 0.16) × 10^3^	1.56 ± 0.09	(1.56 ± 0.09) × 10^4^
10^4^	4.81 ± 0.31	(4.81 ± 0.31) × 10^3^	5.08 ± 0.44	(5.08 ± 0.44) × 10^4^	1.55 ± 0.07	(1.55 ± 0.07) × 10^5^
10^3^	4.48 ± 0.90	(4.48 ± 0.90) × 10^4^	5.13 ± 0.01	(5.13 ± 0.01) × 10^5^	1.60 ± 0.01	(1.60 ± 0.01) × 10^6^
10^2^	1.86 ± 0.71	(1.86 ± 0.71) × 10^5^	5.51 ± 0.25	(5.51 ± 0.25) × 10^6^	1.65 ± 0.13	(1.65 ± 0.13) × 10^7^
10^1^	0.94 ± 0.08	(0.94 ± 0.08) × 10^5^	3.12 ± 0.73	(3.12 ± 0.73) × 10^7^	1.45 ± 0.01	(1.45 ± 0.01) × 10^8^

* *Number of bacteria at the moment of deviation of the microcalorimetric power-time curves obtained in liquid and in renneted reconstituted skim milk samples*.

**Table 4:** **Mean radii of the colonies of *Streptococcus thermophilus* ST12 in r-RSM at the “deviation moment,” at the end of exponential growth phase and at the end of cultivations at different inoculation rates 10^6^–10^1^ cfu mL^−1^**.

**Inoculation rate, cfu mL^−1^**	***R*col at power-time curves “deviation moment,” μm**	***R*col at the end of exponential phase, μm**	***R*col at the end of fermentation (at 22 h), μm**
10^6^	2.55, 0.01	5.22, 0.02	7.33, 0.01
10^5^	4.94, 0.33	11.16, 0.11	15.95, 0.31
10^4^	10.14, 0.10	23.69, 0.68	33.85, 0.51
10^3^	22.29, 0.36	51.21, 0.02	73.72, 0.22
10^2^	38.33, 0.92	113.05, 1.69	160.40, 4.13
10^1^	64.68, 3.07	200.73, 15.77	331.12, 1.08

*Radii of average colonies were calculated from average volumes of the colony determined using Kepler's conjecture of bacterial packing of colonies*.

It can be seen from the Table 3 that as expected from the measurements of the heat the total numbers of bacteria in samples at different inoculation rates were practically equal at the end of the cultivations, which automatically means that the grown colonies contained different number of cells. The same was true for the data from the end of the exponential growth.

In agreement with the same type of logic the radii of average colonies at the end of fermentation were varying from 7 μm at the highest inoculation rate 10^6^ CFU mL^−1^ to 331 μm at the inoculation rate 10^1^ CFU mL^−1^ (Table 4). Average size of colonies at inoculation rate 10^5^ CFU mL^−1^ after 22 h of fermentation was similar to that measured in a model cheese by Floury et al. (2013)—average diameters of colonies being approximately 30 μm and 30–50 μm, respectively.

It should be emphasized that only one-third of biomass was synthesized by the end of the second exponential growth phase in r-RSM whereas most of the biomass was synthesized during the post-exponential growth phase (Table 3). In contrast to that radii of the colonies at the ends of the second exponential phases reached approximately 70% of the final values.

If the data obtained showed that the mechanisms determining the end of the exponential phase and end of cultivation in our experiments at 22 h were not depending on the inoculation rates, then the mechanism leading to the “deviation moment” is more complicated. As seen from the Tables 3, 4 neither the total numbers of bacteria N^*^ nor the sizes of average colonies were equal at different inoculation rates at the moment when the growth rate of bacteria decreased in the r-RSM in comparison with that in RSM.

### Utilization of carbohydrates and production of lactic acid by the bacteria in r-RSM

The studies of consumption of lactose and formation of organic acids were carried out in the case of inoculation rate of 10^5^ CFU mL^−1^.

**Figure 4:**
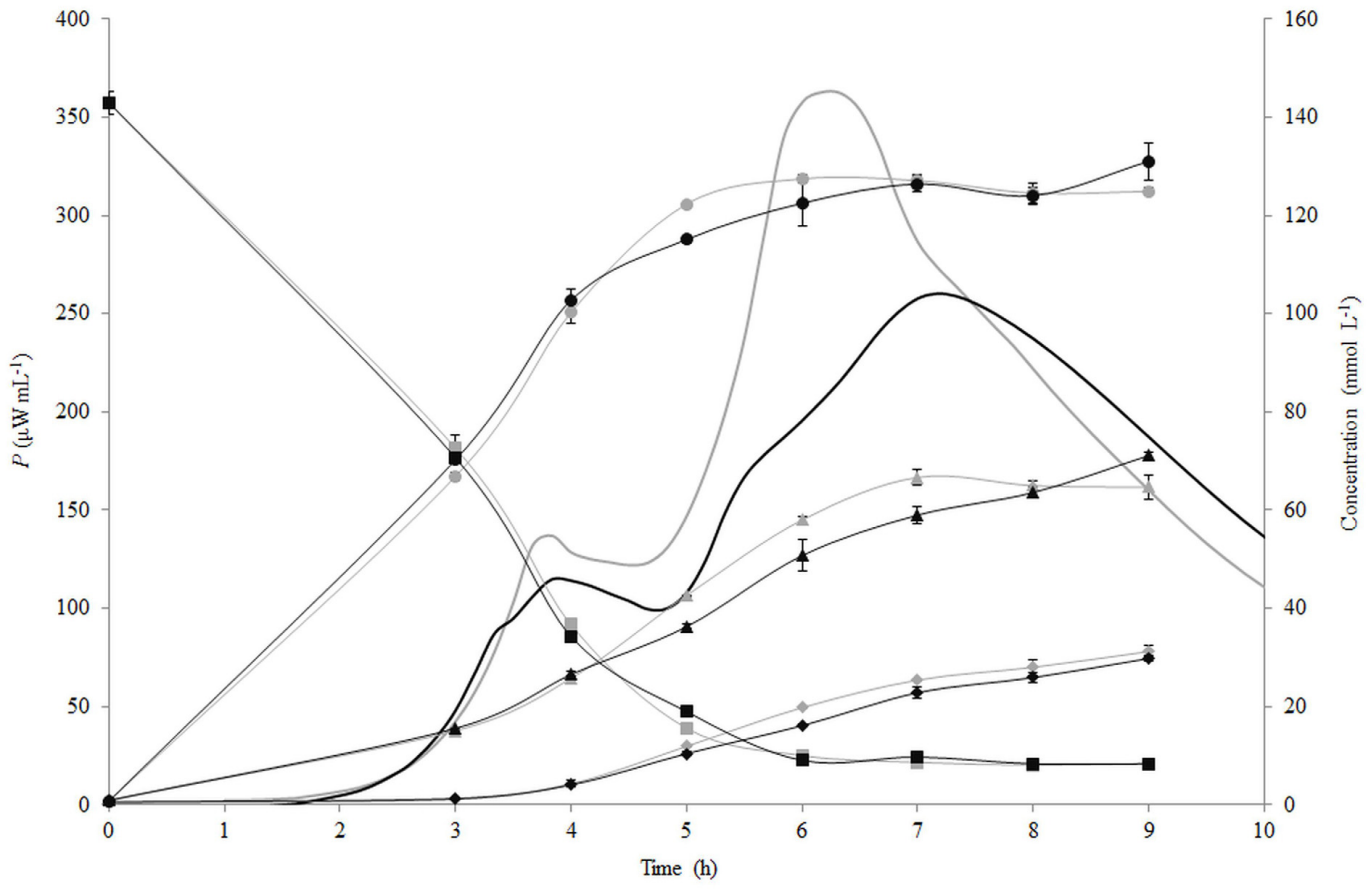
**Calorimetric power-time curves (bold line), lactose (■), glucose (●), galactose (▲) and lactic acid (◆) profiles during the growth of *Streptococcus thermophilus* ST12 in liquid (gray) and renneted (black) reconstituted milk**.

The changes of the concentration of carbohydrates (lactose, glucose, galactose) and lactic acid during fermentation of RSM and r-RSM with *St. thermophilus* ST12 at inoculation rate 10^5^ CFU mL^−1^ are presented in Figure 4. As seen from this figure, the patterns of hydrolysis of lactose, release of glucose and galactose and formation of lactic acid were practically identical in both RSM and r-RSM (Figure 4). Approximately 130 mmol L^−1^ of lactose was hydrolysed at the end of the second exponential growth phase (during active growth), 127 mmol L^−1^ of glucose and 58 mmol L^−1^ of galactose was utilized by the bacteria, and about 20 mmol L^−1^ of lactic acid was produced.

**Figure 5:**
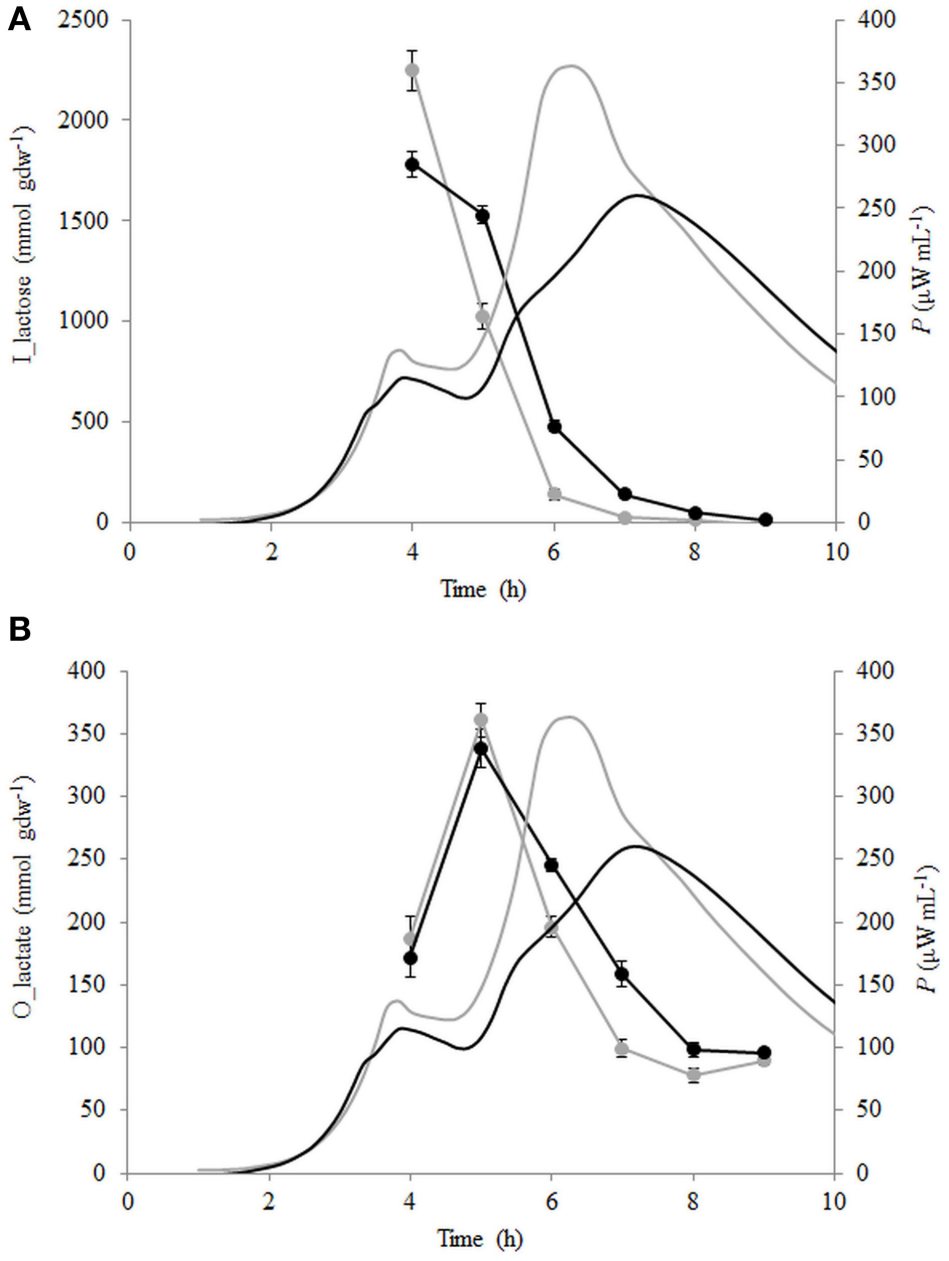
**Calorimetric power-time curves (bold line), lactose consumption (A) and lactate production (B) per biomass formed (mmol gdw^−1^) of *Streptococcus thermophilus* in liquid (gray) and renneted (black) reconstituted milk**.

Lactose consumption and lactate production per biomass formed (mmol gdw^−1^) were calculated (see Section Calculation of Growth Parameters Characterizing Metabolism of the Growing Cells) and are presented in Figures 5A,B. During the growth of *St. thermophilus* ST12, lactose was consumed very fast—up to 2200 and 1800 mmol gdw^−1^ in liquid and renneted RSM, respectively. Lactose consumption led to the accumulation of galactose, as well as glucose (Figure 5), due to low glycolytic activity of the bacteria, reflected by the low lactate yield per lactose below 0.2 mol mol^−1^ in RSM as well as in r-RSM (theoretical maximum 4 mol mol^−1^). It can be assumed that galactose was not metabolized by this strain; however, the accumulation of galactose was lower than predicted compared to the consumption of lactose. This indicated that either galactose was consumed through glycolysis, or it was used for polysaccharide production. Usually polysaccharides are produced in growth-restricted conditions to synthesize carbon storage substances for the future.

### Change of free amino acids during fermentation in r-RSM

**Figure 6:**
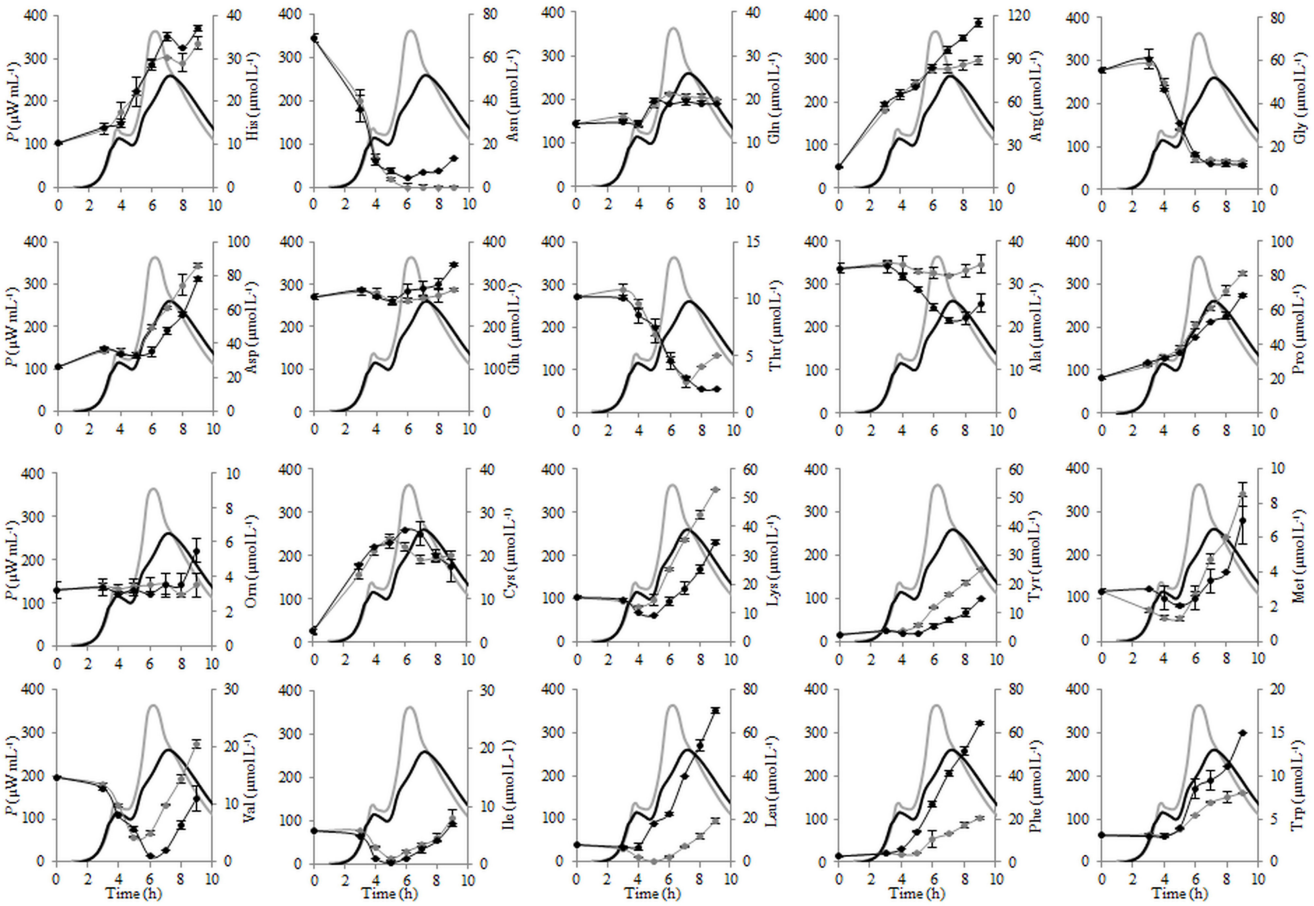
**The changes of the concentrations of the individual free amino acids (FAA) during the growth of *Streptococcus thermophilus* ST12 at inoculation rate 10^5^ CFU mL^−1^ in RSM (gray) and r-RSM (black)**.

The changes of the concentrations of the individual FAA during fermentation of liquid and renneted RSM with *St. thermophilus* ST12 at inoculation 10^5^ CFU mL^−1^ were evaluated and presented in Figure 6. As seen from Figure 6, glutamic acid was initially the most abundant free amino acid in both, RSM and r-RSM, accounting for 46% of the total.

**Figure 7:**
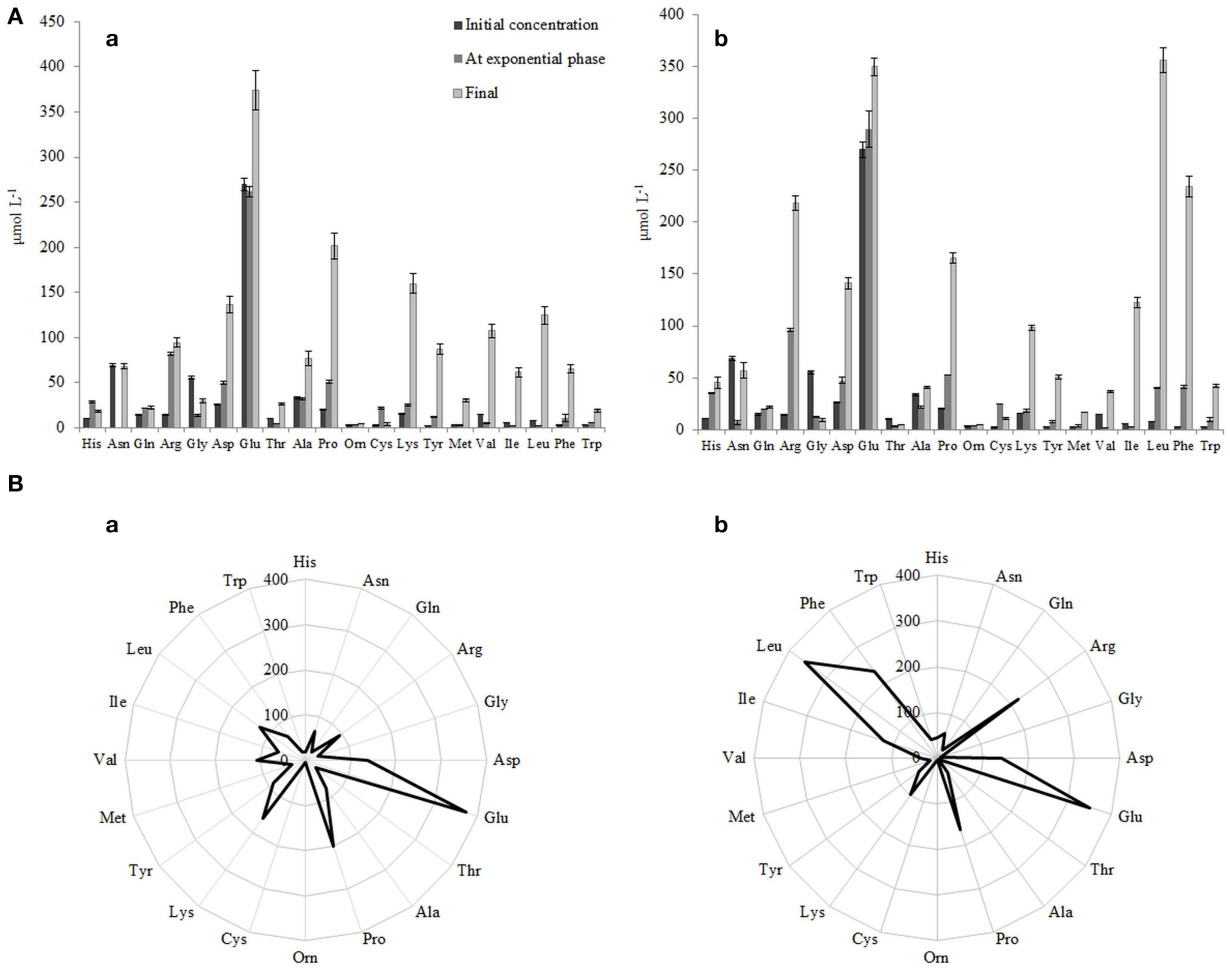
**(A)** Initial concentrations of free amino acid (FAA) and concentrations of FAA at the end of the exponential phase and at the end of growth in RSM (a) and r-RSM(b). **(B)** Radar-diagram showing the concentrations of FAA at the end of growth (at 22 h) in RSM (a) and r-RSM (b).

There was a dramatic reduction of asparagine, glycine and valine at the end of exponential phase in both RSM and r-RSM. Leucine showed a clear decline in RSM during fermentation, whereas in the case of r-RSM a larger increase of this amino acid as well as phenylalanine was observed. It can be seen from the data presented in Figure 7 that the intensive liberation of the majority of amino acids in RSM and r-RSM was observed during the period from the end of exponential phase till the end of fermentation (from 5 h till 22 h).

**Figure 8:**
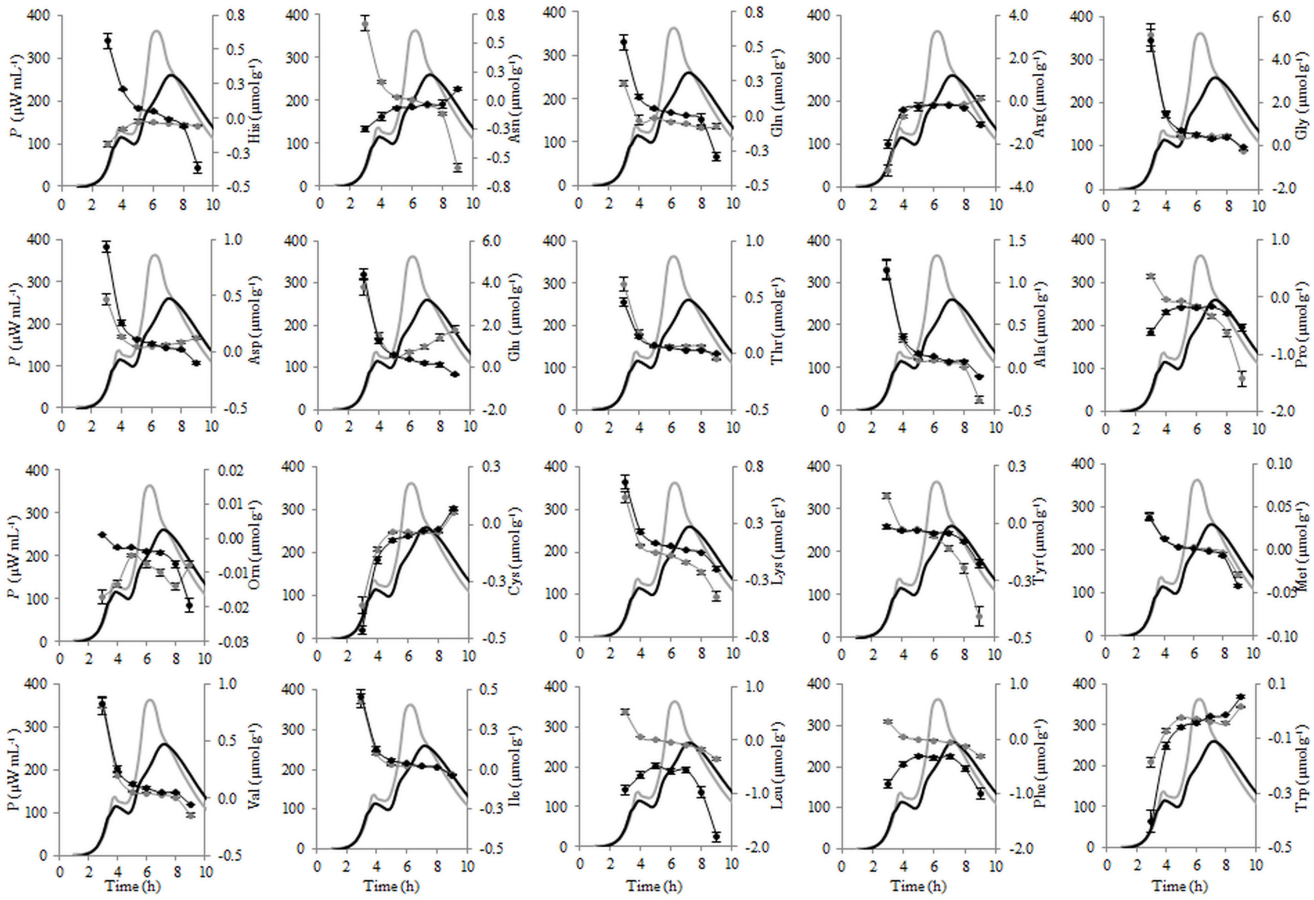
**Calorimetric power-time curves (bold line) and the consumptions (line with circles) of the individual FAA during the exponential growth of *Streptococcus thermophilus* ST12 at inoculation rate 10^5^ CFU mL^−1^ in RSM (gray) and r-RSM (black)**.

The concentrations of the total free amino acids (TFAA) were 15% higher in r-RSM than those determined in RSM at the end of cultivations, 2024 and 1713 μmol L^−1^, respectively. Glutamic acid, proline, lysine and aspartic acid were the most predominant FAA in RSM at the end of fermentation (at 22 h), accounting for 22, 12, 9, and 8% of the total, respectively. In contrast, leucine, glutamic acid, phenylalanine, arginine and proline, accounting for 18, 17, 12, 11, and 8% of the total were the dominant amino acids in r-RSM—see Figure 8.

### Metabolism of amino acids and growth of the bacteria

Proteolysis in rennet curd is catalyzed by enzymes from coagulant and enzymes from the inoculated *St. thermophilus* The initial hydrolysis of caseins is caused by the chymosin which results in the formation of large (water-insoluble) and intermediate-sized (water-soluble) peptides which are degraded subsequently by the coagulant and enzymes from the starters (Sousa et al., 2001). The final products of proteolysis are FAA and their concentration in curd is the net result of the liberation of amino acids from casein, their degradation to catabolic products and also utilization and synthesis by the starter bacteria. Free amino acid concentrations changes were evaluated in liquid and renneted milk samples during the growth of *St. thermophilus* ST12—see Figures 6–8. Amino acid consumption during the 1st and 2nd exponential growth phases is presented in Table 5 Although *St. thermophilus* has not been shown to be auxotrophic for any single amino acid, growth is abolished when both Glu/Gln and Cys/Met are removed (Letort et al., 2002). In addition, the exhaustion of Leu and Val reduces the growth rate more than three times and only six amino acids (Asn, Ala, Ile, Gly, Ser, and Thr) can be individually omitted without any effect on the growth rate.

**Table 5:**
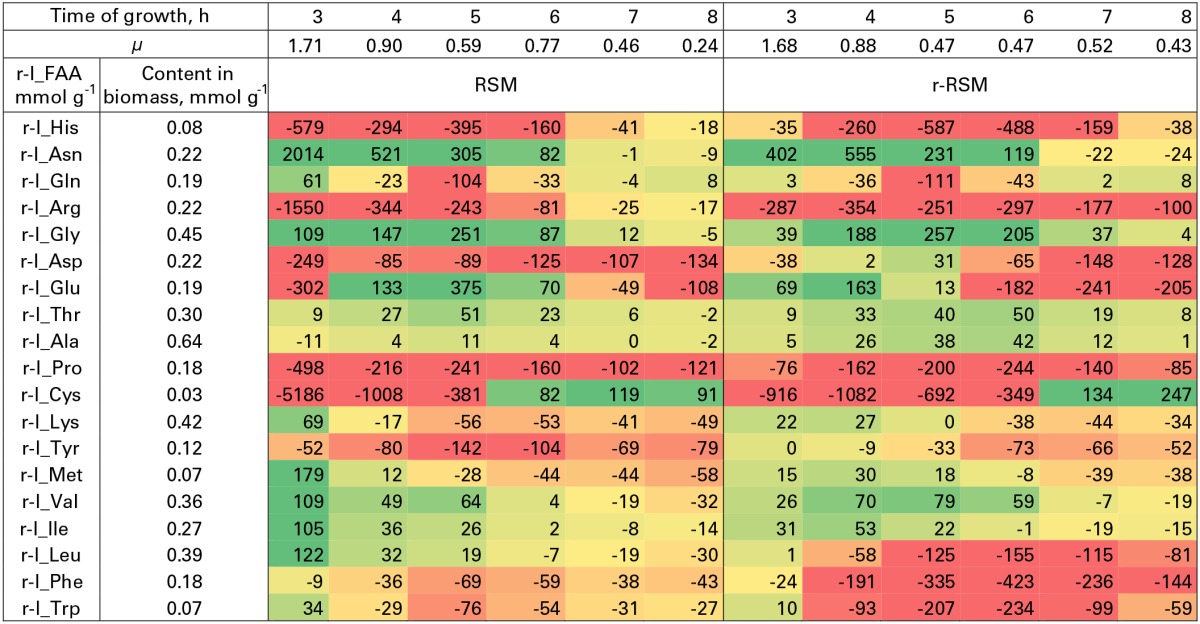
**Relative consumption of free amino acids (r-I_FAA) compared to that required for the synthesis of biomass proteins (%)**.

*Green color shows that free amino acids were consumed more than required for biomass synthesis. Amino acid composition of biomass is taken from the literature (Adamberg et al., 2012)*.

Most of the trends in consumption of FAA during the exponential growth of *St. thermophilus* ST12 in RSM and r-RSM were similar—see Tables 5, 6. The only amino acids consumed in amounts corresponding to the calculated requirements for biomass synthesis in the 1st exponential phase were Gly, Glx (Gln+Glu) and Asx. These amino acids were also the only amino acids whose concentrations in milk exceeded the amount required for biomass synthesis (Table 6). The amount of free His was also sufficient to satisfy biomass requirements, but it should be noted that the amount of this amino acid did not decrease during the exponential growth of the bacteria. Most of the concentrations of other FAA could not support the growth of cells more than 30%. Free Arg and Pro in milk could support amino acid requirements for over 40% but instead of decrease the concentrations of these amino acids increased during the 1st exponential growth phase. This pattern was quite general. Accumulation of amino acids in the media, especially in renneted milk samples due to their release from proteins, and most noticeably during the second exponential growth phase, made it impossible to analyse consumptions of amino acids in this growth phase.

**Table 6:**
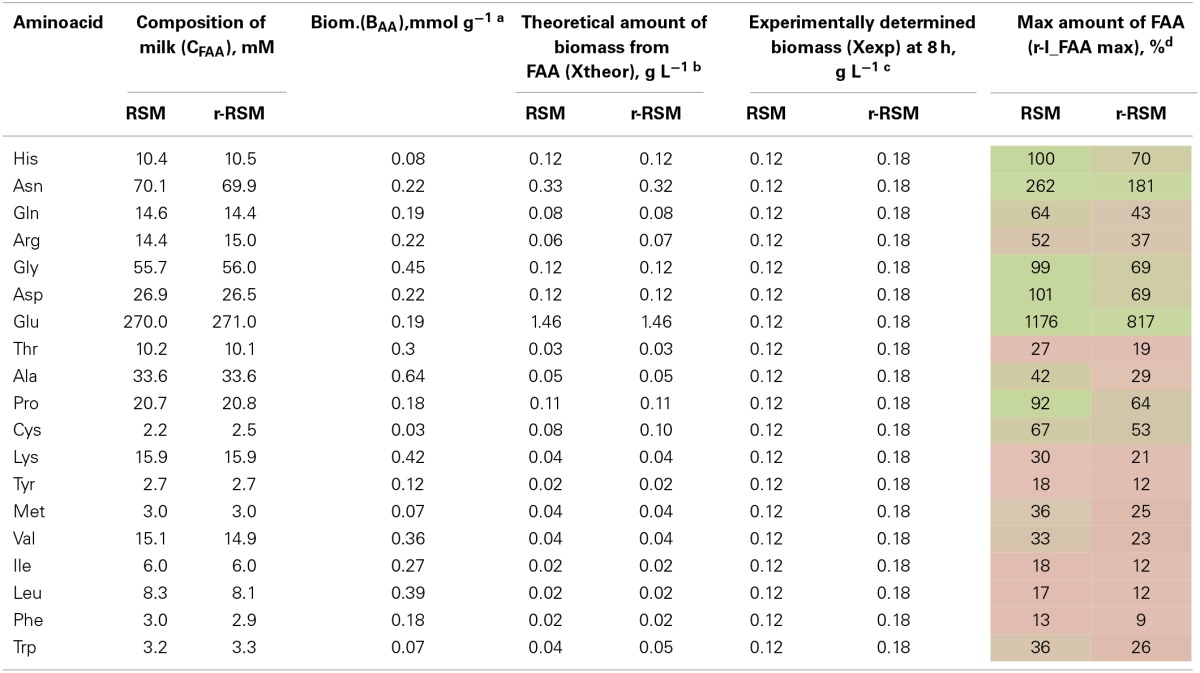
**Milk composition, maximal amount of biomass that can be obtained from free amino acids (FAA) in milk and relative amount of FAA supporting the synthesis of biomass proteins**.

*^a^Biomass composition is taken from literature (Adamberg et al., 2012)*.

*^b^Calculated as X_theor_ = (C_AA_/1000)/B_AA_ indicating how much biomass can be produced from free amino a acid present in milk assuming that amino acid is used only for the synthesis of biomass proteins. AA refers to corresponding amino acid*.

*^c^Experimentally determined biomass concentration assuming that heat produced per cell is 4.45 × 10^−9^ J per cell and cell mass is 0.2 × 10^−12^ g*.

*^d^Calculated as r-I_FAA max = X*_theor_ × *100/X*_exp_ *indicating relative amount of amino acid in biomass proteins that can be supported by consumed free amino acids. Percentage below indicates that free amino acid was less consumed than required for synthesis of biomass proteins*.

Overconsumption of Glu 5–7 times exceeding the need for biomass synthesis indicated the potential of the synthesis of other amino acids from it. In total, 4.6 mmol g^−1^ of amino acids were incorporated into the biomass. Of the total, 30% was covered by the consumption of FAA (excluding Glu). If we take into account Glu overconsumption and amino group transfer to other amino acids, Glu can provide ammonia for 25% of amino acids of the biomass. The remaining 45% of amino acids must be have been derived from peptides.

## Discussion

During the last years, there has been a significant increase of interest in study of growth of bacterial colonies in dairy food matrices (especially in cheese matrices). It has been shown that location, distribution of the cells in different zones of the matrices as well as size of the colonies and distances between colonies are important factors determining peculiarities of cheese ripening processes, influencing utilization of the nutrients and diffusion of metabolites within the cheese matrices (Ercolini et al., 2003; Jeanson et al., 2011; Floury et al., 2013). However, the first systematic comparative study of quantitative peculiarities of growth and metabolism of a LAB *St. thermophilus* ST12 at different inoculation rates in liquid and renneted milk during the whole growth of the bacteria was carried out in this paper to our knowledge. A novel non-invasive method of microcalorimetry developed by us was used throughout the study. The method developed and used allowed obtain the first time quantitative data on the growth kinetics of colonies of different sizes in renneted milk.

It was shown that the growth patterns of *St*. *thermophilus* ST12 in r-RSM during fermentation at 40°C were clearly multiphase in comparison with diauxic growth of *St. thermophilus* ST12 in RSM. Decrease of the maximal calorimetric growth rates *μ_max_* during the first and the second exponential growth phases as well as remarkably lower values of maximum heat flows *P*_max_ (W mL^−1^) of the thermophilic starters growing in r-RSM were observed. The noted inhibition of growth rates in the r-RSM could be explained most probably by the accumulation of lactate in the colonies leading to the change of pH. This conclusion was supported also by the fact that lactate inhibition was not observed in RSM where the buffering capacity of liquid milk was sufficient for alleviating of the influence of lactate (see Figure 3).

The data obtained showed that the numbers of bacteria in the end of the exponential phase (combined exponential phase, see above), as well as in the end of the cultivations were practically the same at different inoculation rates, see Table 3. This allowed introduce a very simple calculation scheme for the determination of the sizes of the colonies, including the radii, in these important points at different inoculation rates presented in Table 3. However, the total numbers of bacteria in the end of the exponential growth in r-RSM were higher than in RSM for about 13% at all except at low inoculation rates. This fact could be explained by the lower rate of decrease of pH in the r-RSM in comparison with RSM indicating that buffering capacity of the matrices is important in determining the end of the exponential growth of the bacteria in milk, not accumulation of lactate directly.

It should be noted that the total numbers of bacteria in the “deviation points” were not the same at different inoculation rates as expected from the data presented above. This was indicating that these points were determined not by a “uniform” mechanism like the end of exponential growth phases and the end of growth during the cultivation but in this case most probably also the sizes of colonies were playing more important role in determining the local buffering capacity in the colonies. Another factor derived from the size of colonies is the availability of nutrients (essential amino acids) for bacteria as proteases are cell bound and cannot freely move in the environment to deliberate peptides from casein.

An observation showing that the growth rates of bacteria in small colonies in the beginning of the cultivation at all inoculation rates were the same as in liquid milk indicated that diffusion rate of glucose was practically the same in these two types of samples.

## Conclusions

The present study clearly showed potential of non-invasive method of microcalorimetry in combination with the other analytical methods in study of quantitative peculiarities of growth and metabolism of bacteria in opaque and solid state media—see a detailed comparison of the methods in Lobete et al. (in press). It was possible to elucidate the quantitatively characterized growth patterns of *Streptococcus thermophilus* ST12 in renneted milk at different inoculation rates and compare them with the patterns of growth of the bacteria in milk. Clear discriminative results of growth and metabolic patterns of cells in renneted and liquid milk encourage use of microcalorimetry more widely as a screening tool for strain selection in their natural environment (milk or curd).

### Conflict of interest statement

The authors declare that the research was conducted in the absence of any commercial or financial relationships that could be construed as a potential conflict of interest.
